# Protective Roles of DMP1 in High Phosphate Homeostasis

**DOI:** 10.1371/journal.pone.0042329

**Published:** 2012-08-03

**Authors:** Afsaneh Rangiani, Zhengguo Cao, Yao Sun, Yongbo Lu, Tian Gao, Baozhi Yuan, Anika Rodgers, Chunlin Qin, Makoto Kuro-o, Jian Q. Feng

**Affiliations:** 1 Biomedical Sciences, Baylor College of Dentistry, Texas A&M Health Science Center, Dallas, Texas, United States of America; 2 Medicine, University of Wisconsin and GRECC, Madison, Wisconsin, United States of America; 3 Pathology, University of Texas SW Medical Center, Dallas, Texas, United States of America; 4 The State Key Laboratory Breeding Base of Basic Science of Stomatology (Hubei-MOST) & Key Laboratory of Oral Biomedicine, Ministry of Education, Department of Periodontology, School & Hospital of Stomatology, Wuhan University, Wuhan, China; UAE University, United Arab Emirates

## Abstract

**Purpose:**

*Dmp1* (dentin matrix protein1) null mice (*Dmp1^−/−^*) display hypophosphatemic rickets with a sharp increase in fibroblast growth factor 23 (FGF23). Disruption of Klotho (the obligatory co-receptor of FGF23) results in hyperphosphatemia with ectopic calcifications formed in blood vessels and kidneys. To determine the role of DMP1 in both a hyperphosphatemic environment and within the ectopic calcifications, we created *Dmp1/Klotho* compound deficient (*Dmp1^−/−^kl/kl*) mice.

**Procedures:**

A combination of TUNEL, immunohistochemistry, TRAP, von Kossa, micro CT, bone histomorphometry, serum biochemistry and Scanning Electron Microscopy techniques were used to analyze the changes in blood vessels, kidney and bone for wild type control, *Dmp1^−/−^*, *Klotho* deficient (*kl/kl*) and *Dmp1^−/−^kl/kl* animals.

**Findings:**

Interestingly, *Dmp1^−/−^kl/kl* mice show a dramatic improvement of rickets and an identical serum biochemical phenotype to *kl/kl* mice (extremely high FGF23, hyperphosphatemia and reduced parathyroid hormone (PTH) levels). Unexpectedly, *Dmp1^−/−^kl/kl* mice presented elevated levels of apoptosis in osteocytes, endothelial and vascular smooth muscle cells in small and large blood vessels, and within the kidney as well as dramatic increase in ectopic calcification in all these tissues, as compared to *kl/kl*.

**Conclusion:**

These findings suggest that DMP1 has an anti-apoptotic role in hyperphosphatemia. Discovering this novel protective role of DMP1 may have clinical relevance in protecting the cells from apoptosis in high-phosphate environments as observed in chronic kidney disease (CKD).

## Introduction

Physiologically, phosphate (Pi) is critical not only for the health of mineralized tissues such as bone and teeth, but is also essential for a variety of biological processes, including energy metabolism, cell signaling, nucleic acid synthesis, and membrane function [1]. Pathologically, low blood Pi levels lead to hypophosphatemic rickets [2], [3]; whereas high blood Pi levels, even in the upper-to-normal range, have been linked to increased mortality within populations of both general and chronic kidney disease (CKD) [4]. To maintain Pi homeostasis, parathyroid hormone (PTH) and 1, 25-dihydroxy vitamin D (1,25 (OH)_2_D_3_) play an important role via their regulation of phosphate absorption in the intestines and reabsorption in the kidney [5]. Recent findings suggest that FGF23 is a more specific and potent phosphaturic hormone [6], [7], [8]. FGF23, mainly expressed in bone, targets the kidney to remove phosphate with little effect on calcium homeostasis [9], [10]. *Fgf23* null mice display increased renal phosphate reabsorption and increased serum 1,25 (OH)_2_D_3_ levels, which results in hyperphosphatemia, hypercalcemia, and vascular calcification [10].

DMP1, an extracellular matrix phosphoprotein highly expressed in hard tissues such as the skeleton and teeth, belongs to small integrin ligand N-linked glycoprotein family [11], [12]. Deletion of *Dmp1* in mice or mutations in DMP1 in humans causes hypophosphatemic rickets with an elevated circulating level of FGF23, which is responsible for the impaired renal tubular reabsorption of phosphate [13], [14]. The abnormalities observed in *Dmp1* null mice include decreased endochondral ossification, defects in bone lengthening and remodeling, increase in the width of the bone (flaring), and an increased metaphysis area [15], [16], [17], [18]. A number of these characteristics are related to the direct role of DMP1 as a regulator of osteocyte maturation [19], whereas other characteristics are secondary to impaired Pi homeostasis [8].

Klotho, a transmembrane protein predominantly expressed in the kidney, was initially identified as an anti-aging gene prior to µµ the discovery of FGF23 during which a loss-of-function study in mice showed a striking aging phenotype encompassing hair loss, infertility and emphysema [20]. Interestingly, *Fgf23* null mice unexpectedly exhibited almost an identical aging phenotype as that of *Klotho*-deficient (*kl/kl*) mice [10]. On the other hand, *kl/kl* mice display high serum levels of phosphate, calcium, and vitamin D, which is very similar to that of the *Fgf23* null phenotype [21]. Furthermore, mechanistic studies demonstrated that FGF23 requires not only the FGF receptors (FGFRs), but also Klotho, as FGF23 is able to bind to FGFRs with high affinity only in the presence of Klotho. This suggests that Klotho is an obligatory co-receptor of FGF23 [22]. One of the major complications in *kl/kl* mice is the high frequency of formation of ectopic calcifications in the blood vessels and kidney. This is assumed to be the consequence of either high serum phosphate levels [23], cell death that occurs in CKD patients [24], or the matrix vesicles released from the dead vascular smooth muscle cells [25].

As DMP1, a key regulator of FGF23 [13], [14], is also expressed in blood vessels and kidney tissues [26], [27], we explored the potential (beneficial or harmful) role of DMP1 in soft tissue calcification within *Dmp1* ^−/−^ *kl/kl* compound mice lacking both *Klotho* and *Dmp1*. This model displayed high phosphate retention and increased FGF23 levels which resemble chronic kidney disease (CKD). We also attempted to test whether elevation of blood Pi levels due to Klotho deficiency would rescue the rickets phenotype in *Dmp1^−/−^* mice. Our data support a novel concept that DMP1 plays an important role in blocking ectopic calcification in *Klotho* deficient mice via its anti-apoptotic function within a high phosphate environment.

**Figure 1:**
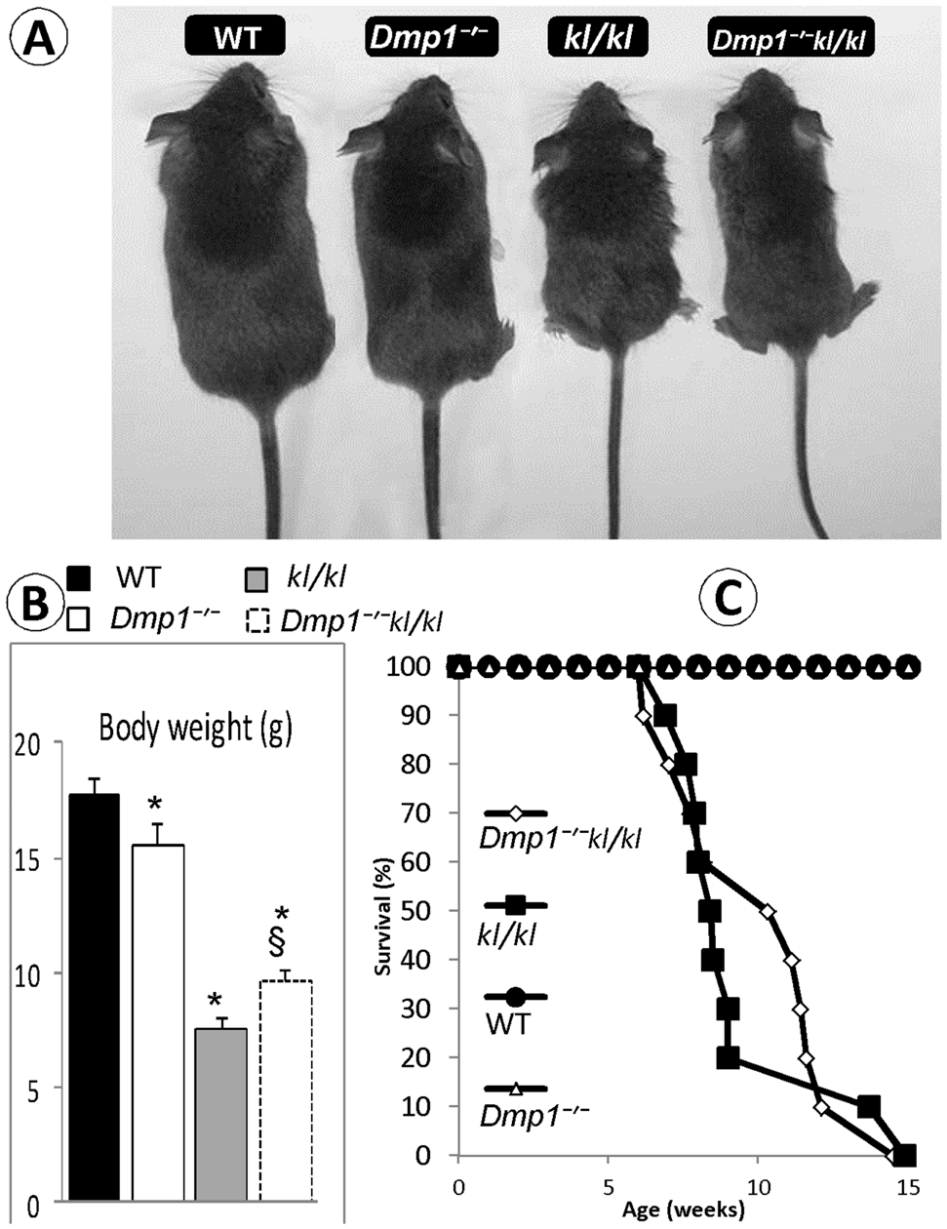
Macroscopic phenotype of *Dmp1/Klotho* compound deficient mice (*Dmp1^−/−^kl/kl*). (A) Gross appearance of Wild Type (WT), Dmp1 null *(Dmp1^−/−^)*, Klotho deficient *(kl/kl)*, and compound deficient *(Dmp1^−/−^kl/kl)* mice at 6 weeks of age. (**B**) Body weights of 6 weeks old mice, showing a decrease in *Dmp1^−/−^* (13%), *kl/kl* (57%) and *Dmp1^−/−^kl/kl* (36%) mice compared to the age matched WT (P = 0.02; P<0.001, and P<0.001 respectively; n = 10 in each group). Note that the *Dmp1^−/−^kl/kl* body weight was ∼ 30% higher than that of the single *kl/kl* mice (P = 0.03). (**C**) Survival for WT, *Dmp1^−/−^, kl/kl*, and *Dmp1^−/−^kl/kl* mice (n = 10 in each group). Both *kl/kl* and *Dmp1^−/−^kl/kl* mice showed similar low survival rate compared to WT and *Dmp1^−/−^* groups. About 40% of *Dmp1^−/−^kl/kl* animals survived 3 weeks more than single *kl/kl*. Neither of the kl/kl nor *Dmp1^−/−^kl/kl* groups survived more than 15 weeks. None of WT and *Dmp1^−/−^* animals died in 30 weeks of observation period. These mice were of similar genetic backgrounds but not necessarily littermates. *P<0.05 compared to WT; § P<0.05 compared to *kl/kl*.

**Figure 2:**
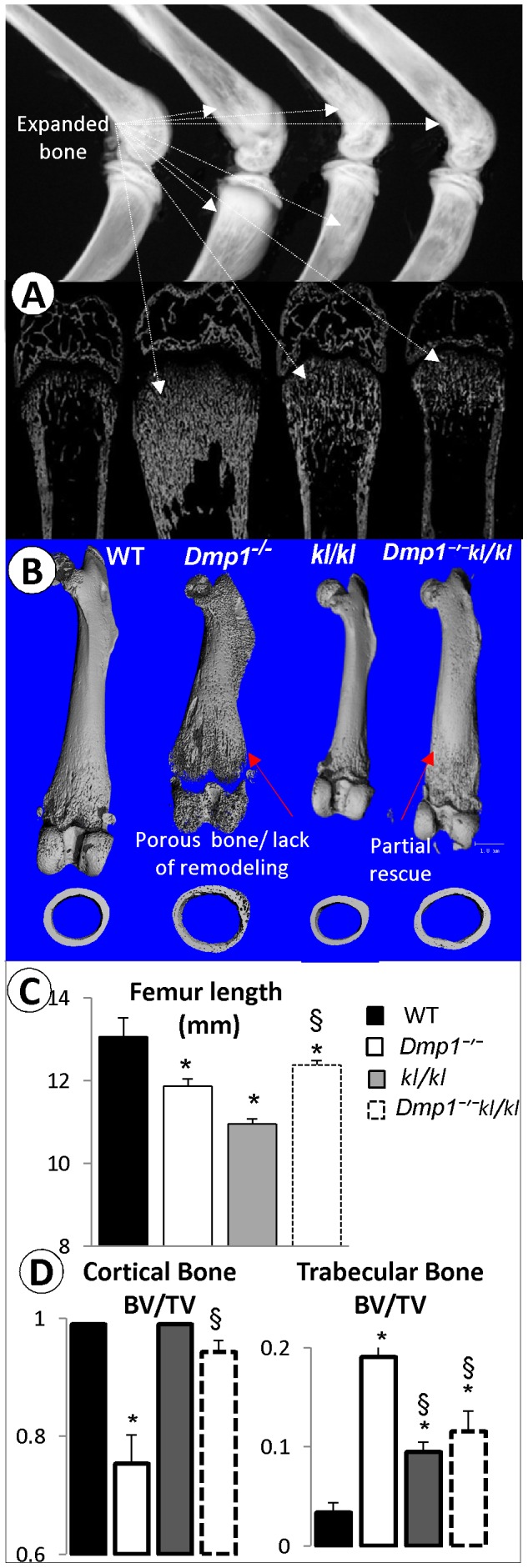
The changes of bone volume/mineral content (reflected by BV/TV) and femur length in *Dmp1^−/−^*, *Klotho* deficient (*kl/kl*) and the compound deficient (*Dmp1^−/−^ kl/kl*) at age of 6-weeks. (**A**) Representative X-ray (upper panel) and backscattered SEM (lower panel) images of the hind limbs obtained from wild type (WT), *Dmp1^−/−^*, *kl/kl* and *Dmp1^−/−^ kl/kl* mice. (**B**) Representative µCT images of the above four group femurs at the longitudinal front view (upper panel), and the midshaft cross section view (lower pane). (**C**) Quantitative µCT data shows moderate changes of femur length in *Dmp1^−/−^*, *kl/kl* and *Dmp1^−/−^kl/kl* mice compared to the age-matched WT control (P<0.001, P<0.001, and P = 0.03 respectively; n = 10). Note that the femur length difference between the *Dmp1^−/−^kl/kl* mice and *kl/kl* (P<0.001) or *Dmp1^−/−^* (*P<0.05) are significant. (**D**) The Quantitative µCT data show a sharp reduction of BV/TV in the *Dmp1^−/−^* cortical bone (P<0.01), and there is no significant change between WT and *kl/kl* or the *Dmp1^−/−^kl/kl* (left panel). In contrast, there is a significant increase in metaphysis BV/TV in *Dmp1^−/−^* (>80%, P<0.01) or in the *kl/kl* (>50%, P<0.01) or in the *Dmp1^−/−^kl/kl* mice (>60%, P<0.01) separately (right panel).

## Materials and Methods

### Ethical Approval

All mice were maintained under guidelines established by Baylor College of Dentistry Institution of Animal Care and Use Committee (IACUC). IACUC has specifically given ethical approved for all the procedures in this study.

### Animals

*Klotho*-deficient mice (*kl/kl*) and *Dmp1* lacZ knock-in null mice (*Dmp1^−/−^*) were described previously [20], [28]. The *kl/kl* mouse was originally described as a severe hypomorph strain because extremely low levels of Klotho mRNA were detectable only by RT-PCR [20]. However, Klotho protein was undetectable in any tissues by immunoblot, immunoprecipitation, and immunohisotchemical analyses (data not shown). In addition, *kl/kl* mice were shown to exhibit the same phenotypes as Klotho−/− mice [29]. Thus, the *kl/kl* mouse is virtually equivalent to a null strain. All the mice were fed with tap water and autoclaved Purina rodent chow (5010, Rastlon Purina) containing calcium, 0.67% phosphorus and 4.4 international units of vitamin D/g.

**Figure 3:**
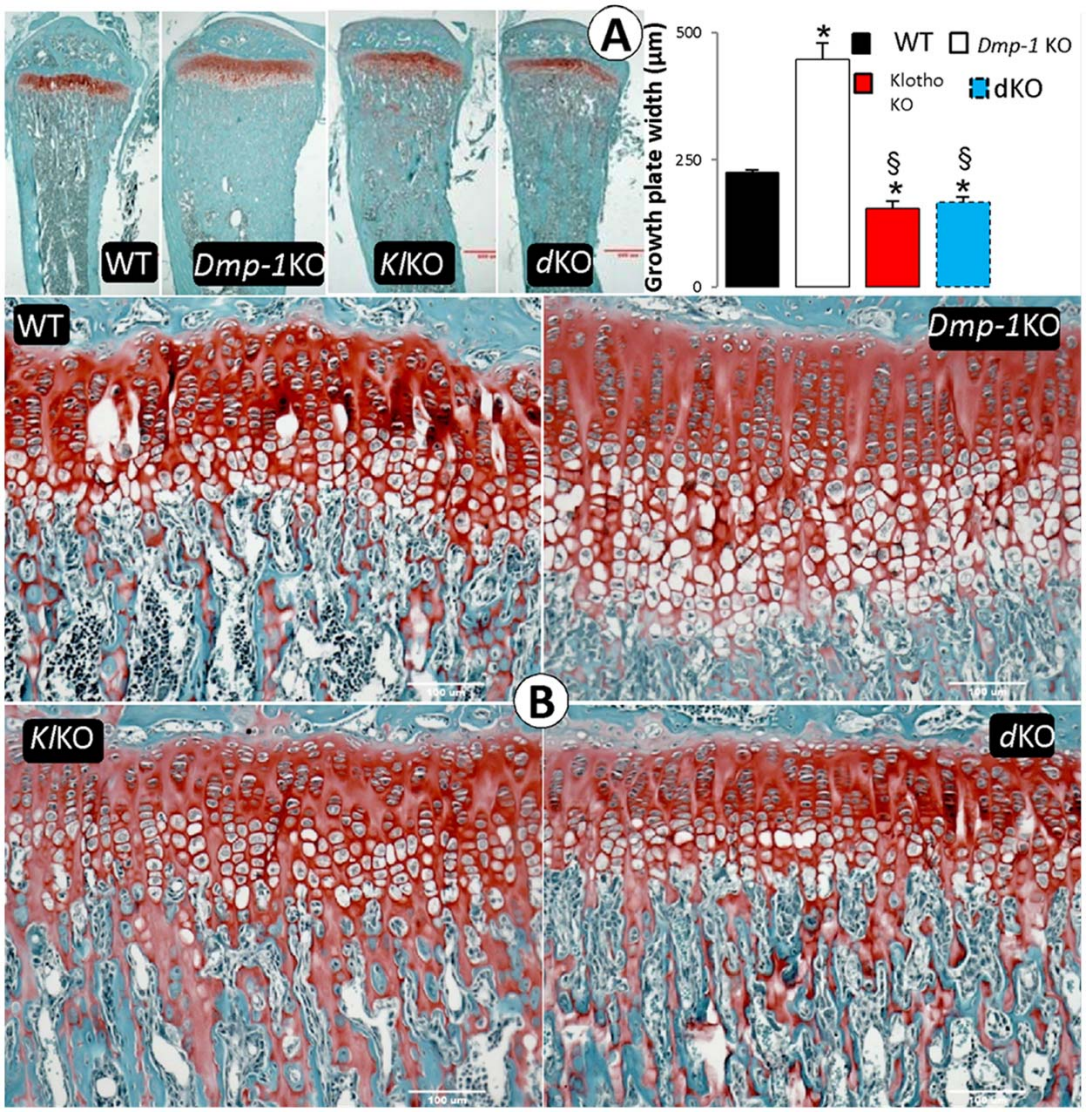
The effect of deletion of *Klotho* on histological features of the *Dmp1^−/−^* growth plate and metaphysis area. (**A**) Growth plates and metaphysis of proximal tibias obtained from WT, *Dmp1^−/−^*, *kl/kl*, and *Dmp1^−/^kl/kl* (Safranin O staining x200). Note that there is an expansion of metaphysis in the *kl/kl*, which is similar to that of the *Dmp1^−/−^* and *Dmp1^−/−^kl/kl* mice. (**B**) growth plate width comparison between all four groups of the mice shows increase of phosphate in both *kl/kl* and *Dmp1^−/−^kl/kl* mice resulted in decreased growth plate width compared to WT (P = 0.009 and 0.024 respectively). Values reported in mean ± SE from more than 3 mice in each group at 6 weeks of age. *P<0.05 compared to WT; § P<0.05 compared to *Dmp1^−/−^* group.

**Figure 4:**
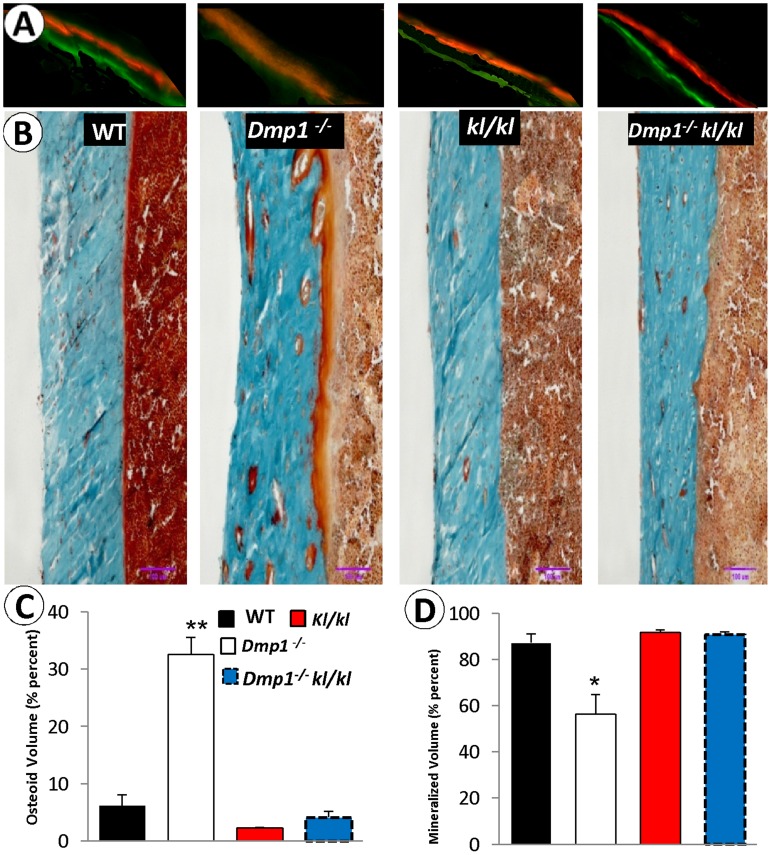
Ablation of *Klotho* rescued bone formation rate and osteomalacia phenotype in the compound deficient (*Dmp1^−/−^ kl/kl*) mice. (**A**) Confocal microscope images of flurochrome labeling of 6 weeks old mice tibias (red: alizarin red, green: calcein). Note that in the *Dmp1^−/^kl/kl* mice the bone formation labeling pattern is similar to that of the control and the *kl/kl* mice. (**B**) Goldner’s stain showed the massive osteoid (red) areas commonly seen in the *Dmp1^−/−^* long bone, which was restored to normal in the *Dmp1^−/−^kl/kl* mice. (**C, D**), Quantitative bone histomorphometric analyses showed that the above changes of the osteoid area (**C**) and the mineralized area (blue in color) (**D**) were statistically significant. *P<0.05; **P<0.01 compared to the WT; (n = 3 in each group).

### Generation of *Dmp1/Klotho* Compound Deficient Mice

Both *Dmp1* and *Klotho* genes in mice are located on chromosome 5 (qE5 and qG3 respectively). The generation of *Dmp1/Klotho* compound deficient mice (*Dmp1^−/−^kl/kl*) was based on the chance of cross-over between homologous chromosomes. First, we crossed *Dmp1* ^−/−^ and *Klotho*-deficient heterozygous mice (*kl/+*). Their offspring will include male and female heterozygous mice for both *Dmp1* and *Klotho* (*Dmp1* ^+/−^ *kl/+*) which were set for litter-mating. In the first three generations, no cross-over occurred. After the third generation, *Dmp1* null *Klotho* hetero animals (*Dmp1* ^−/−^ *kl/+*) were born and used for mating with a one-in-four chance for producing compound homozygous mice (*Dmp1^−/−^kl/kl).* At the same time compound hetero mice (*Dmp1^+/−^ kl/+)* were also continued mating to generate wild type (WT), *Dmp1^−/−^* and *kl/kl* animals; A few *Dmp1^−/−^kl/kl* mice were also born through this set of mating.

### Genotyping

DNA was extracted from the toe of each mouse by standard protocol and subjected to PCR for genotyping. *Dmp1^+/−^ and Dmp1^−/−^* were genotyped by PCR as reported previously [28]. For *Dmp1*, the PCR program consisted of 4 minutes initial denaturation at 94°C, amplification cycle including denaturation at 94°C for 1 minute, annealing at 55°C for 2 minutes, and extension for 3 minutes at 72°C, and final extension was performed at 72°C for 10 minutes. The expected product size for the targeted *Dmp1* allele was 280 bp and the wild type allele was 410 bp. The *Klotho* genotyping protocol and the primers used have been previously mentioned [30]. Briefly for *Klotho* genotyping, Takara LA Taq and buffer were used and the protocol for PCR included 2 minutes initial denaturation at 94°C, amplification cycle consisting denaturation at 94°C for 30 seconds, annealing at 56°C for 30 seconds, and extension for 1.5 minute at 72°C, which was followed by final extension at 72°C for 10 minutes. The expected product size for WT *Klotho* was 458 bp and for mutant *Klotho* was 920 bp.

**Figure 5:**
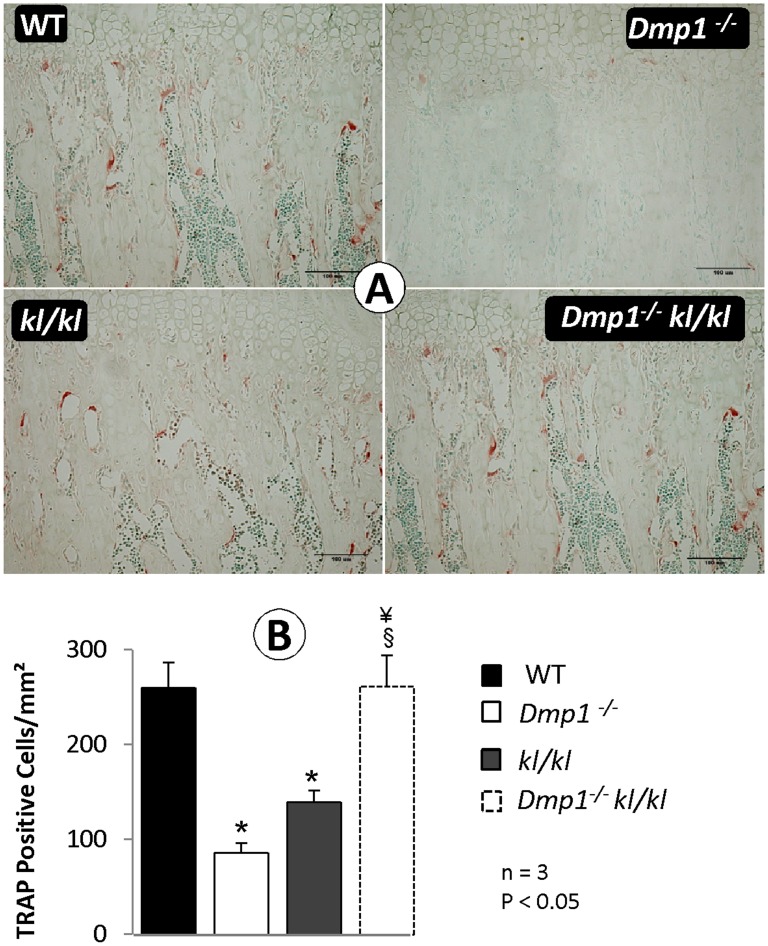
The compound deficiency of *Klotho* and *Dmp1* increases osteoclast number to the normal level (6 weeks old mice). **(A**) TRAP-stained metaphysis of the proximal tibias obtained from WT, *Dmp1^−/−^*, *kl/kl* and compound deficient (*Dmp1^−/−^kl/kl*) mice. (**B**) Quantitative analyses of TRAP positive cells per mm^2^ in each group. Values reported in mean ± SE from 3 mice in each group. Osteoclast number in *Dmp1^−/−^* and *kl/kl* bones were significantly lower than WT (P<0.001 for both), while in the *Dmp1^−/−^kl/kl* group it were restored to the same level as of WT. *P<0.05 compared to WT; § P<0.05 compared to *Dmp1^−/−^*; ¥ P<0.05 compared to *kl/kl.*

**Figure 6:**
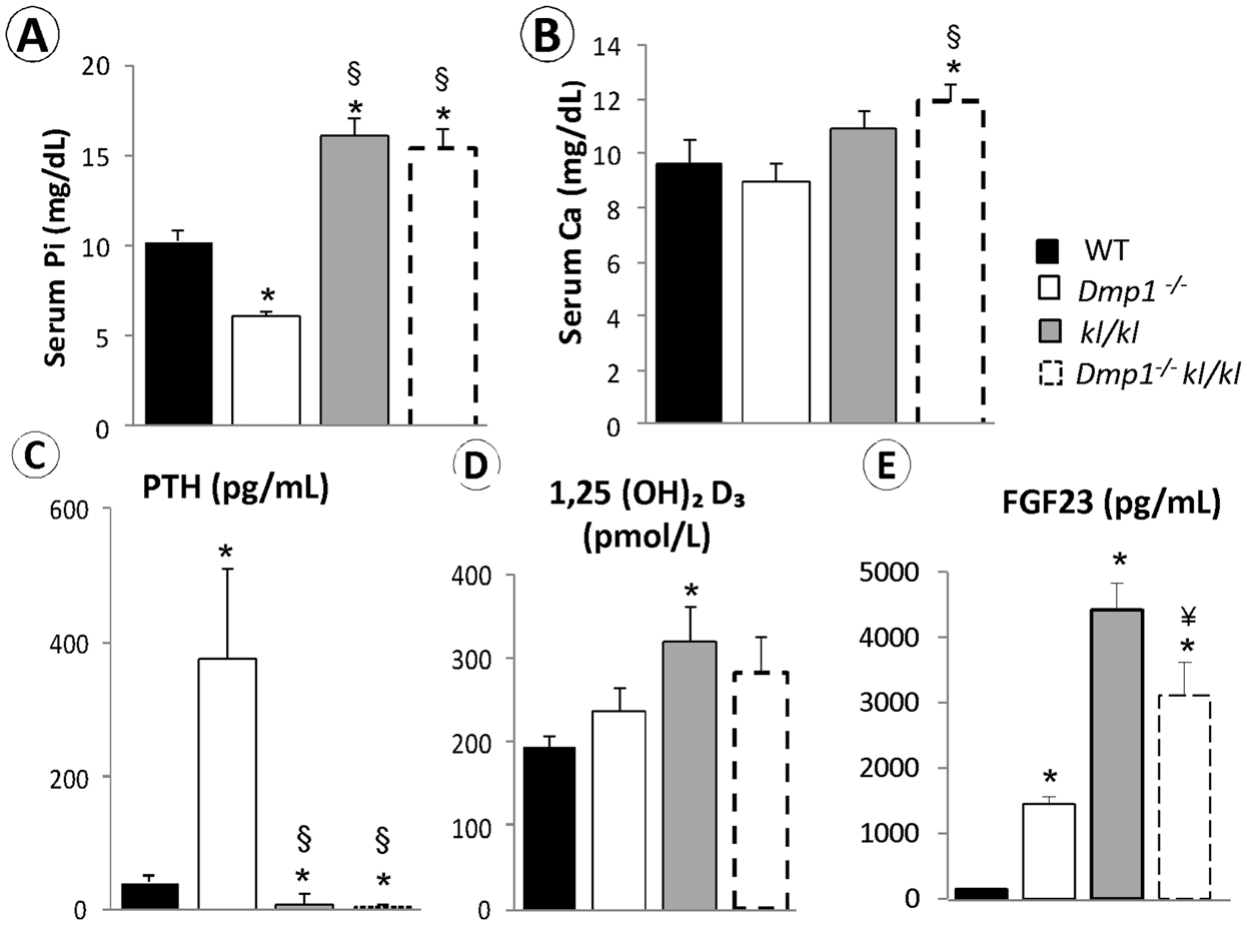
Changes of serum biochemistry profiles in *Dmp1^−/−^*, *kl/kl* and the *Dmp1^−/−^ kl/kl* mice. (**A**) Serum Phosphate levels were significantly lower in *Dmp1^−/−^* mice (P<0.001), while there was a significant increase in *kl/kl* and the *Dmp1^−/−^kl/kl* groups (P<0.001) compared to WT (n = 7 in each group). (**B**) Serum calcium levels were statistically unchanged in *Dmp1^−/−^* and *kl/kl* mice. However, the calcium level was significantly higher in the *Dmp1^−/−^kl/kl* mice than that of the WT (P = 0.04). (**C**) Serum PTH level was over 10 fold increased in *Dmp1^−/−^* but significantly lower in both *kl/kl* and the *Dmp1^−/−^ kl/kl* mice compared to WT (P = 0.025, and P = 0.002 respectively). (**D**) Serum 1,25(OH)_2_D_3_ levels were overall higher in all these deficient mice, although only *kl/kl* mice showed significant difference compared to the WT (P = 0.043). **(E)** Serum FGF23 levels were high in all three deficient mice with *kl/kl* the highest compared to the WT control. *P<0.05 compared to WT, § P<0.05 compared to *Dmp1^−/−^*, ¥ P<0.05 compared to *kl/kl* mice.

### Serum Biochemistry

Blood was collected directly from the heart by using needle for WT, *kl/kl*, *Dmp1^−/−^* and *Dmp1^−/−^ kl/kl* mice before they were sacrificed at 6 weeks of age. Serum was isolated and stored at -80°C. Serum phosphorous, calcium, PTH, vitamin D, and FGF23 levels were measured as described previously [8]. Briefly, the serum phosphorous level was measured by the phosphomolibdate-ascorbic acid method. Serum calcium Controlent analysis was performed using the colorimetric calcium kit (Stanbio laboratory). For measuring serum FGF23, we used full length FGF23 ELISA kit (Kainos Laboratories). PTH serum levels were measured using Mouse PTH 1-84 ELISA Kit (Immutopics, Inc. San Clemente, CA), and the level of 1, 25 hydroxyvitamin D (1, 25 (OH)_2_D_3_) was measured in serum of all four groups of mice using 1,25 Dihydroxy Vitamin D EIA kit (Immunodiagnostic System, Fountain Hills, AZ).

### Histology, Immunohistochemistry and TUNEL Staining

The right tibias of 6 week old mice were collected, fixed in 4% paraformaldehyde in phosphate buffered saline (pH 7.4) for 48 hours, decalcified by microwave EDTA for 16 hours, dehydrated through graded alcohol and then embedded in paraffin. Sections were cut 4.5 µm thick and were mounted on slides and dried. Slides were used for Safranin O and TRAP staining as previously described [11]. Growth plate width was measured for at least 3 mice in each group for Safranin O staining. TRAP positive cells were counted in the area including 300 µm down the growth plate for 3 mice in each group, and the number of the cells was divided by the area for each slide. For immunohistochemistry, mouse anti-alpha rhFGF-23 antibody (Cell Essential) was used on tibias of four study groups including WT, *Dmp1^−/−^*, *kl/kl*, and *Dmp1^−/−^kl/kl* animals. To analyze apoptosis in tissues the TUNEL kit (Roche Diagnostics, IN, USA) was used and the number of apoptotic osteocytes in a 100×500 µm^2^ area was counted in each group. Kidney and aorta samples were fixed and embedded in paraffin without decalcification, and stained for Von Kossa (calcification) and TUNEL (for apoptosis). For undecalcified femurs, samples were embedded in methyl methycrylate, and cut by a Leica 2165 rotary microtome (Ernst Leitz Wetzlar) at a 6 µm thickness. Three samples in each group were stained by Goldner’s masson trichrome staining [8].

**Figure 7:**
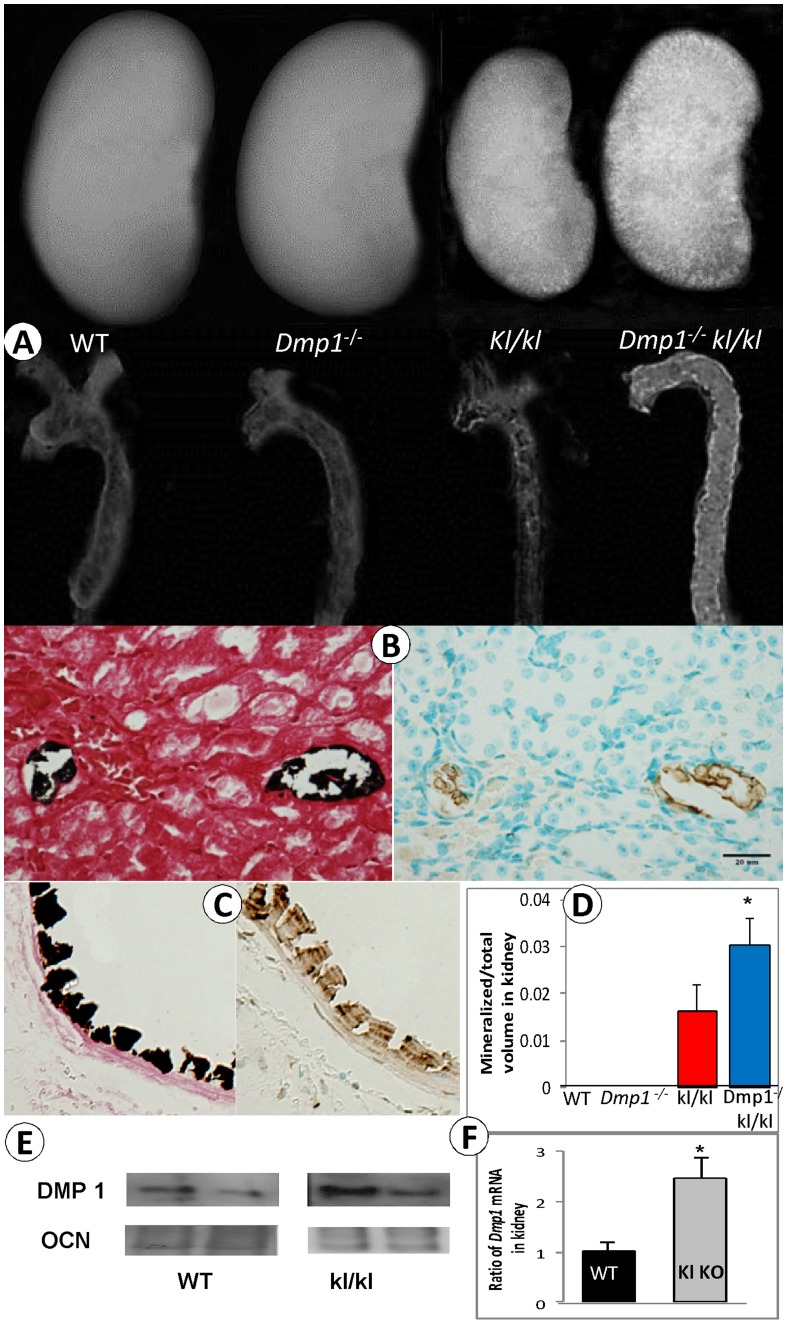
Sharply increased ectopic calcification and apoptosis in the compound deficient (*Dmp1^−/−^ kl/kl*) mice. (**A**) Representative X-ray images of kidney and aorta in four groups show moderate ectopic calcifications in the *kl/kl* kidney and aorta, and massive ectopic calcification in the *Dmp1^−/−^kl/kl* kidney and aorta. (**B**) von-Kossa stain (left panel) and TUNEL assay (right panel) of the *Dmp1^−/−^kl/kl* kidney showing a close correlation between the calcified- and apoptotic cells. (**C**) A similar linkage between the ectopic calcification (von-Kossa stain; right panel) and the apoptosis (TUNEL assay; right panel) was observed in the adjacent *Dmp1^−/−^kl/kl* aorta. (**D**) The quantitative mineral content is sharply increased in both the *kl/kl* and the *Dmp1^−/−^kl/kl* kidney by µCT analysis with significantly higher in the *Dmp1^−/−^kl/kl* kidney than that of the single kl/kl kidney (P = 0.041). Values are means ± SEM of 3–5 kidneys. Note that there was no calcification in the WT and the *Dmp1^−/−^* mice. (**E**) Representative DMP1 Western blots with WT at left panel and *kl/kl* at right panel shows higher density of DMP1 in *kl/kl.* Osteocalcin (OCN) is the matrix protein which does not show difference between these two groups. (**F**) Quantitative kidney RT-PCR data showing over two fold-increases of *Dmp1* mRANA in *kl/kl* compared to the WT control. n = 3, *P<0.05.

**Figure 8:**
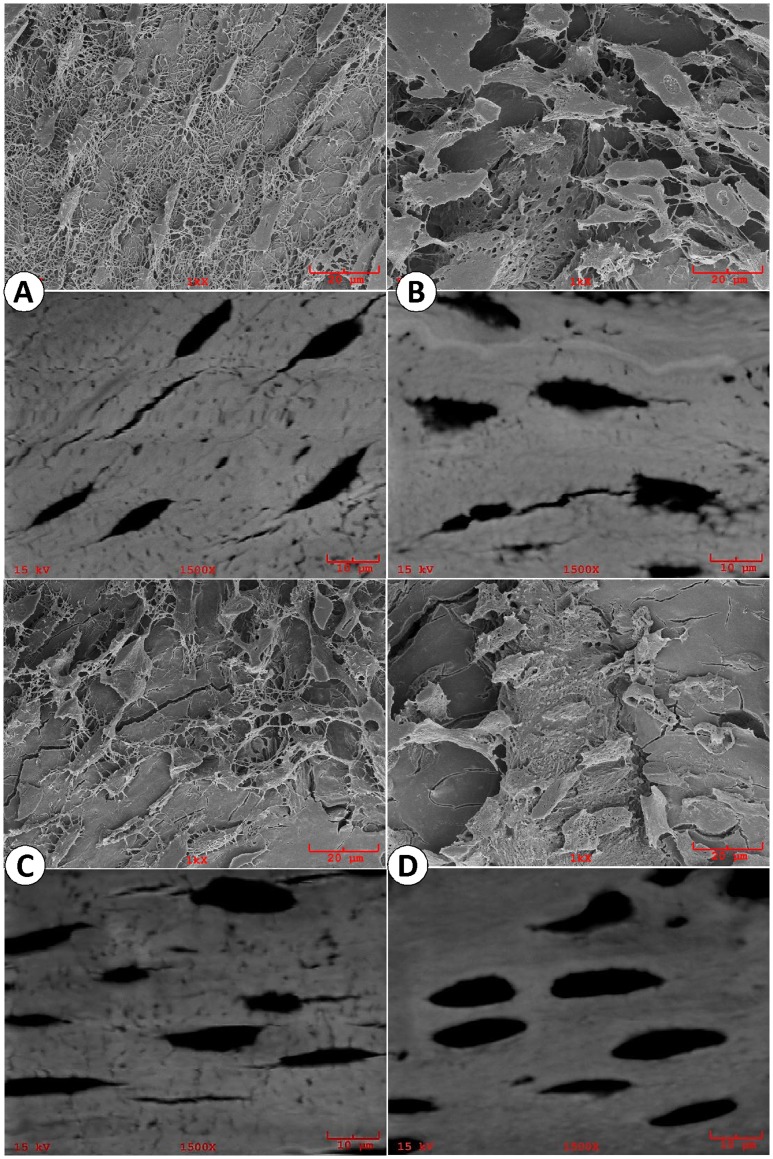
Acid-etched resin casting SEM (upper panels) and backscattered SEM (lower panels) images of osteocytes in four animal groups. (A). Representative SEM images showed well organized WT osteocytes which were generally straight and run perpendicular to the long axis of the osteocyte with numerous dendrites; (B). The *Dmp1^−/−^* osteocyte appeared much larger in size, and the distribution of the osteocytes were less organized with a sharp reduction in the dendrite number; (C). The *kl/kl* osteocytes were less straight and more randomly oriented with moderate reduction in osteocyte dendrites; (D). The *Dmp1^−/−^kl/kl* osteocytes, much larger in size, showed absence of dendrites with poor organization in their cell distributions.

### Microcomputed Tomography (µCT), Radiography, and Sacnning Electron Microscopy (SEM)

After the fixation, femurs were dissected and radiographs was taken using Faxitron radiographic inspection unit (Model 8050, Field Emission Corporation, Inc), with digital capture image capability.

Femur cortical and trabecular compartments were quantified using an X-ray microCT imaging system (µCT 35, Scanco Medical, Basserdorf, Switzerland). Serial tomographic imaging at an energy level of 55 kV and intensity of 145 µA for the femurs was performed. To analyze the trabecular region, we used 500 cross sectional slides in a constant area under the growth plate in all samples. The area was determined as 100 slides lower than the lowest part of growth plate. In addition, cortical thickness data was obtained at the midshaft of the bone. For this purpose, cortical bones from 100 cross sectional slices above the midshaft of the femur were analyzed. The threshold used for this analysis was 335 for trabecular bone and 283 for cortical bone. Micro CT imaging system was also used to evaluate and compare the calcification level in kidneys of all four groups of mice. Bone volume/total volume of each group was calculated and used for comparison.

To image the osteocyte lacunocanalicular system, SEM of resin casted bone samples was performed. Bone tissues were fixed in 70% ethanol and embedded in MMA (Buehler, Lake Bluff, IL, USA). The surface of the MMA-embedded bone was polished, followed by acid etching with 37% phosphoric acid for 2 to 10 seconds, 5% sodium hypochlorite for 5 minutes, and coating with gold and palladium. Samples were examined by an FEI/Philips XL30 field emission environmental SEM (Phillips, Hillsboro, OR, USA). For Backscattered Electron Microscopy imaging we used the method described previously [17].

### Bone Histomorphometry

Using bioquant software, the analysis of osteoid and mineralized bone was performed on the cortical bone area of tibias of the four groups of mice. The identification of mineralized and osteoid area was based on their color in Goldner’s masson trichrome staining; orange to red represents osteoid, and blue represents well mineralized bone. The ratio of mineralized bone or osteoid on total volume of the bone is reported in percent.

### Fluorochrome Labeling of the Mineralization Front

Compound fluorescence labeling was performed to visualize the bone mineralization rate in all four groups of *WT, Dmp1^−/−^, kl/kl* and *Dmp1^−/−^kl/kl* as described previously [31]. Briefly the first intraperitoneal injection was performed using calcein green (5 mg/kg), followed by injection of an alizarin red label (5 mg/kg i.p.; Sigma- Aldrich) 5 days later. Mice were sacrificed after 48 hours. The tibias were collected and fixed in 70% ethanol for 48 hours. Without decalcification the samples were dehydrated through a graded series of ethanol (70% to 100%) and embedded in methyl methacrylate. Then 15-mm sections were cut using a Leitz 1600 saw microtome (Ernst Leitz Wetzlar GmbH, Wetzlar, Germany). The unstained sections were viewed under epifluorescent illumination using a Nikon (Melville, NY, USA) E800 microscope.

### Primary Cell Culture for Osteoblasts and Colorimetric TUNEL Assay

Calvarial cells, enriched for cells with an osteoblast phenotype, were isolated from 3 day old *Dmp1^−/−^* pups and age-matched control pups using procedures described previously [28]. Briefly, the first-passage cells were plated at density of 2×10^4^ cells/well in 96-well tissue culture dishes (Costar, Corning, NY, USA). Cells were grown to confluence in α-MEM supplemented with 10% FBS. The medium then was changed to α-MEM supplemented with 10% FBS only (normal media), or supplemented with 1 or 5 mM of phosphate added to normal media. After 24 hours of incubation with the new media, cells were fixed in 4% PFA for 7 minutes and washed in PBS. To evaluate and compare apoptosis between the different groups, HT Titer TACS colorimetric assay kit (Trevigen, Gaithersburg, MD, USA) was used. Briefly cells were treated with Cytonin for 30 minutes and after wash with dH_2_O. After incubation with hydrogen peroxidase, TdT labeling buffer was used for 5 minutes. Then samples were incubated with stop buffer followed by adding Strep HRP solution was to samples for 10 minutes. Samples were washed and incubated by Saphire solution for 30 minutes in dark. Reaction stopped by 0.2 N HCl and absorbance measurement was done at 450 nm. For the cell proliferation assay, Cell Counting Kit-8 was used. Cell proliferation was assessed according to the instruction of the CCK-8 kit (BI Yuntian Co, China). In brief, after incubation with normal media for 24 hours, 10 µL WST-8 was added to each well, and the plates were incubated for an additional 4 hrs at 37°C to convert WST-8 into formazan. The absorbance of each plate was measured at 450 nm (absorbance) with spectrophotometer. The absorbance presents as OD450 nm represents a direct correlation with the cell number in the well.

### RT-PCR and Real Time PCR (qPCR)

For real time PCR (qPCR), the total RNA was extracted from mouse tissues in both the kidney and humerus at 6 weeks of age. To evaluate the net expression of FGF23 mRNA in osteocytes, the osteoblastic layer on the surface and bone marrow were both removed. Total RNA was isolated from the kidneys, and cortical bone using TRI-reagent (Molecular Research Center, Cincinnati, OH, USA) as described previously [11]. First-strand cDNA was synthesized from the kidney RNAs using iScript cDNA Synthesis kit (SA Biosciences, Frederick, USA). The 20 µL reverse transcriptase reaction was based on 1 µg total RNA. The iCycler iQ Real-Time PCR Detection System and iQ SYBR Green Supermix (Agilent Technologies, USA) were used for real-time quantitative PCR analysis. The expression was normalized by glyceraldehyde-3-phosphate dehydrogenase (GAPDH) in the same sample and expressed as 100% of the control (WT). The forward primer sequence used for qPCR for the *Dmp1* gene is 5 ′AGTGAGTCATCAGAAGAAAGTCAAGC 3′ and the reverse primer is 5′ CTATACTGGCCTCTGTCGTAGCC 3′.

### Western Blot

Total protein from the kidney was extracted by 5ml extraction reagent (4M guanidine hydrochloride and 0.5 M EDTA) followed by centrifugation (20 min, 10000 rpm, 4°C). The supernatant protein solution was then collected and went through a buffer change procedure (to the final concentration at 0.1M NaCl/6M Urea). The urea-protein buffer was loaded to the Q-sephorose column in Fast Protein Liquid Chromatography (FPLC) for acidic proteins (such as DMP1) separation with a gradient buffer elution range at 0.1–0.8M NaCl/6 M urea. Next, Stains-All staining was used to profile DMP1 and other acidic proteins in all the chromatographic fractions. For Western immunoblotting, anti-DMP1-C-857 and osteocalcin [32] was used at 1∶1000 for one hour. Blots were washed three times in PBS containing 0.3% Tween 20, followed by incubation in the alkaline phosphate-conjugated anti-rabbit IgG (Sigma Aldrich; Louis, MO) at a dilution of 1∶5000. Finally, the blots were incubated with the chemiluminescent substrate CDP-Star (Ambion; Austin, TX) for 5 min and exposed to X-ray films [33].

### Statistics

The differences were evaluated among groups by one way ANOVA, with Benferoni correction and P<0.05 was considered as statistical significance. The values are reported in mean ± SE. For statistical analysis we used SPSS software.

**Figure 9:**
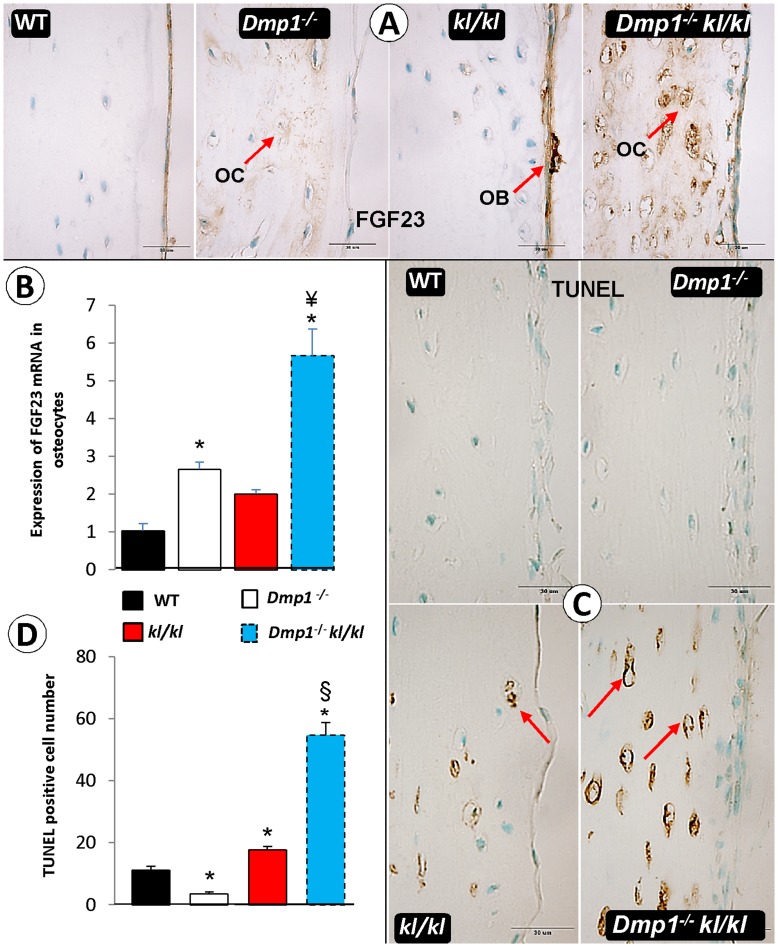
The effect of Ablation of *Dmp1* in *Klotho* deficient background increases apoptosis and FGF23 expression in osteocytes. (A) FGF23 immunostaining shows in *Dmp1* ^−/−^ and in *Dmp1^−/−^kl/kl* animals the expression of FGF23 is detectable in both osteocytes (OC) and osteoblasts (OB), while in *kl/kl* mice it is mainly expressed in osteoblasts with much higher density compared to WT. (B) Quantitative real time PCR on bone after removal of osteoblast layer in *Dmp1^−/−^* shows increased FGF23 expression compared to WT (P = 0.016), but this expression was significantly higher in *Dmp1^−/−^kl/kl* osteocyte (P<0.001). (C) TUNEL staining on four groups of mice shows dramatic increase in double deficient mice (*Dmp1^−/−^kl/kl*) even compared to single *kl/kl* in osteocytes. (D) Counting apoptotic cells in 500 µm^2^ of slides shows *Dmp1^−/−^* has significantly lower rate of apoptosis compared to WT (P = 0.027), and this rate is higher in both *kl/kl* and the *Dmp1^−/−^kl/kl* mice (P = 0.05 and P<0.001 respectively). Ablation of *Dmp1* in *Dmp1^−/−^kl/kl* mice increased apoptosis rate in osteocytes even compared to single *kl/kl* (P<0.001). (OC, osteocyte; OB, osteoblast).

## Results

### Compound *Dmp1 Klotho* Deficient Mice Display an Improvement in Growth Retardation

From a gross overview, there was no apparent difference between *Dmp1* ^−/−^ and wild type (WT) control mice except for a mild reduction in body weight (**Fig. 1A–B**). The body weight of both the *kl/kl* and compound deficient mice was significantly lower than that of the WT mice. The body weight of the compound deficient mice was slightly improved compared to that of the *kl/kl* mice, likely due to an increase in skeleton length and ectopic ossification after a switch from hypophosphatemia to hyperphosphatemia. Consistent with previously reported literature [20], [21], [34] *kl/kl* mice showed a distinct premature aging phenotype, including emphysema, generalized atrophy of the skin, spleen, intestine, skeletal muscle, reproductive organs, and infertility, which was also evident in *Dmp1^−/−^kl/kl* animals (data not shown). However, none of these phenotypes were observed in *Dmp1* ^−/−^ mice.

Compared to the age matched WT mice, both *kl/kl* and *Dmp1^−/−^kl/kl* mice survived for a shorter length of time than WT mice, and neither *kl/kl* nor *Dmp1^−/−^kl/kl* mice survived longer than 15 weeks (**Fig. 1C**). In contrast, there was no difference between the *Dmp1* ^−/−^ and WT mice regarding their survival rate (**Fig. 1C**). Interestingly, about 80% of *kl/kl* mice died before the age of 9 weeks, while 60% of *Dmp1^−/−^kl/kl* mice survived longer than 12 weeks, suggesting that ablation of *Dmp1* in *kl/kl* background improves the life span of *kl/kl* mice (**Fig. 1C** **)**.

### Ablation of *Klotho* Partially Rescued Rickets Features in *Dmp1^−/−^* Mice

The classical characteristics of the rickets phenotype in *Dmp1^−/−^* mice, including the expanded growth plate, enlarged diaphysis, and shorter long bone length have been previously reported [17], and it was proposed that both hypophosphatemia and the direct local role of DMP1 are responsible for these pathological changes [2], [8], [19]. Here we show a dramatic improvement of the osteomalacia and rickets phenotypes in *Dmp1^−/−^kl/kl* compared to *Dmp1^−/−^* mice at 6-weeks of age through the use of radiography, backscattered SEM (**Fig. 2A**) and µCT imaging (**Fig. 2B**). Quantitative data displayed a moderate but significant improvement in the *Dmp1^−/−^kl/kl* femur length when compared to the *Dmp1^−/−^* femur (**Fig. 2C**). Note that there was over a 25% reduction of bone volume/total volume (BV/TV) (reflecting relative changes in bone volume density) in the *Dmp1^−/−^* cortical bone compared to the age-matched control (P<0.01, **Fig. 2D**, *left panel*), which was restored in the *Dmp1^−/−^kl/kl* cortical bone with no significant difference compared to the WT control (P = 0.125). Interestingly, there was a significant increase in the BV/TV in *Dmp1^−/−^* metaphysis (>80%) and *kl/kl* metaphysis which was also present in *Dmp1^−/−^kl/kl*. Taken together, our x-ray, backscattered SEM, and µCT data showed that there is a significant improvement of BV/TV in the compound long bone. This improvement, plus the restoration of the growth plate and bone length in *Dmp1^−/−^kl/kl* mice compared to *Dmp1^−/−^* mice was likely due to a switch from hypophosphatemia to hyperphosphatemia, although the probable local role of Klotho ablation in this phenotypic rescue should not be ignored.

### Removing *Klotho* in *Dmp1^−/−^* Background Improves Histological Abnormalities of the Growth Plate and Cortical Bone Mineralization

To determine the effect of *Klotho* deletion on *Dmp1^−/−^* phenotypes at the histological level, we first confirmed the previous reports [15] showing irregular and disorganized chondrocytes and expanded hypertrophic zone in the *Dmp1* growth plate, which was rescued in *Dmp1^−/−^kl/kl* mice (**Fig. 3A–C**), although the quantitative data displayed a small but significant reduction of the growth plate width in *kl/kl* and *Dmp1^−/−^kl/kl* mice compared to the age matched control (**Fig. 3B**). All these changes (WT, 244±4.8 µm; *Dmp1* ^−/−^, 447±31.6 µm; *kl/kl*, 154.5±14.12 µm; and *Dmp1^−/−^kl/kl*, 166±9.6 µm) are statistically significant. It appears that the identical growth plate phenotype in *kl/kl* and *Dmp1^−/−^kl/kl* is a consequence of a change in Pi homeostasis (see Discussion for details).

Next, we examined changes of mineralization in *Dmp1^−/−^kl/kl* mice using a fluorescent double label assay (alizarin red complexone and calcein). The diffuse fluorochrome labeling pattern commonly observed in *Dmp1^−/−^* mice was restored to a pattern similar to that in both control and single *kl/kl* mice with discrete lines of calcein and alizarin red labeling, suggesting a normal bone-formation rate (**Fig. 4A**). Bone mineralization quality, as reflected by an increase in osteoid area (Goldner stain), is very poor in the *Dmp1^−/−^* long bone, but appears fully rescued in the compound deficient mice as compared to WT and *kl/kl* mice using both qualitative and quantitative analyses (**Fig. 4B–D**).

To address the effect of the *Klotho* deletion on the abnormal bone remodeling commonly observed in *Dmp1^−/−^* mice [19], we examined the number of TRAP (a marker for osteoclastic cells) positive cells, which was sharply reduced in both *Dmp1^−/−^* and *kl/kl* but not in the *Dmp1^−/−^kl/kl* metaphysis at 6 weeks of age (**Fig. 5A**). The above changes were statistically significant (**Fig. 5B**, P<0.05; n = 3). The reduced osteoclast number in *kl/kl* mice may explain in part why there was an increase in the trabecular bone within the metaphysis area (**Fig. 2A**). Interestingly, in *Dmp1^−/−^kl/kl*, the osteoclast number was restored to levels seen in the WT control (**Fig. 5B**). This restoration likely contributed to the improved bone remodeling process in compound deficient mice (**Fig. 2**).

### *Dmp1^−/−^kl/kl* Mice Displayed a Similar Serum Biochemical Profile as that in a Single *kl/kl* Mouse

One of the key differences between *Dmp1^−/−^* and *kl/kl* mice is the Pi level: the former shows hypophosphatemia due to an abnormal increase of FGF23 that leads to Pi waste [8] and the latter is associated with hyperphosphatemia, which is due to a failure of FGF23 to remove extra Pi in the absence of Klotho, the key co-receptor [20], [35]. In addition to its phosphaturic activity, FGF23 suppresses the synthesis of 1, 25 dihydroxyvitamin D_3_ (1,25(OH)_2_D_3_). As low 1,25(OH)_2_D_3_ stimulates PTH production and secretion, there is secondary hyperparathyroidism in *Dmp1^−/−^* mice [8] and PTH suppression in *kl/kl* mice [35]. To clarify whether bone morphological changes as described above are linked to serum biochemical profile changes in these animals, serum levels of Pi, Ca, FGF23, PTH, and 1,25(OH)_2_D_3_ were analyzed. Overall, both *kl/kl* and *Dmp1^−/−^kl/kl* animals shared a similar biochemical profile, including ∼30% increase in Pi, a moderate increase in Ca and 1,25(OH)_2_D_3_, and a suppression of PTH (**Fig. 6A–D**). Surprisingly, the FGF23 level in *Dmp1^−/−^kl/kl* mice was between the levels of *Dmp1^−/−^* and *kl/kl* mice (**Fig. 6E**).

### *Dmp1^−/−^kl/kl* Mice Display Massive Ectopic Calcifications and Apoptosis in the Aorta and Kidney

*Klotho* deficient mice (*kl/kl*) displayed increased serum levels of 1,25(OH)_2_D_3_ and widespread vascular and kidney calcifications [36], which was completely reversed when the 25-Hydroxyvitamin D3 1-alpha-hydroxylase (1α-hydroxylase, the enzyme which catalyzes the hydroxylation of calcidiol to calcitriol, the bioactive form of Vitamin D) was deleted [37]. It is also known that Pi is essential for the apoptosis of terminally differentiated hypertrophic chondrocytes *in vivo* [17], [38], and that high Pi led to apoptosis in osteoblasts [39] and in endothelial cells *in vitro* [40]. Furthermore, DMP1 was expressed in the blood vessels [26] and the kidneys [41]. To address whether DMP1 plays any role in the ectopic calcification in *kl/kl* mice we first confirmed DMP1-lacZ expression in the blood vessels and the kidney (**Fig. S3–S4**). Next, we examined the kidney and the aorta and observed exacerbated calcification by X-ray images (**Fig. 7A**) and by von Kossa staining (**Fig. 7B–C**). The TUNEL assay showed a close correlation between calcification and apoptosis in endothelial and vascular smooth muscle cells, and a very small number of renal tubules. Calcification and apoptosis in the kidney was observed primarily within small blood vessels in both *kl/kl* and *Dmp1^−/−^kl/kl* mice (**Fig. 7B–C**). Quantitative data further confirmed that the ectopic ossification in the *Dmp1^−/−^kl/kl* kidney was statistically significant (**Fig. 7D**). In contrast, neither WT nor *Dmp1^−/−^* mice displayed ectopic calcification or increased levels of apoptosis (data not shown). The increase in apoptotic cells in the *Dmp1^−/−^kl/kl* mice was confirmed by a Caspase 3 assay and H&E stained empty lacunae (**Fig. S2**). Furthermore, Western Blot and quantitative real time PCR (qPCR) data confirmed that within the *kl/kl* kidneys the expression of DMP1 is higher than that in the WT kidneys, which might be due to a compensatory mechanism protecting against the toxic effect of high Pi. The above data clearly suggests that DMP1 plays an important anti-apoptotic role in hyperphosphatemia, and that deletion of *Dmp1* in *kl/kl* mice leads to exacerbated calcification in the kidney and the aorta.

### Deletion of *Klotho* in the *Dmp1^−/−^* Mice Greatly Accelerated Osteocyte Dendrite Loss

DMP1 is highly expressed in osteocytes, but Klotho is not. Using an acid-etched resin casting SEM technique, which revealed detail morphologies of WT osteocytes (**Fig. 8A**), we confirmed *Dmp1^−/−^* osteocyte abnormalities (including the presence of a rough membrane surface with a sharp reduction of dendrites, **Fig. 8B**) [8], and showed minor changes in *kl/kl* osteocyte dendrite number (**Fig. 8C**). Unexpectedly, there was a much more dramatic change in osteocyte morphology with few osteocyte dendrites remaining (**Fig. 8D**) in *Dmp1^−/−^kl/kl* mice compared to the age matched control. The above data indicates that DMP1 is not only necessary for osteocyte maturation and differentiation as previously reported [8], but also plays a protective role in a high phosphate environment in maintaining a normal osteocyte morphology.

### Unique FGF23 Expression Patterns in *Dmp1^−/−^*, *kl/kl* and *Dmp1^−/−^kl/kl* Bone Cells

Although FGF23 was sharply increased in *Dmp1^−/−^* and *kl/kl* mice, the causes are different in each mouse model: an increase of FGF23 in the former model is primary to Pi retention while the increase in the latter model is secondary to Pi retention. As the primary FGF23 source is osteoblast cells, we first compared FGF23 expression patterns in the four animal groups using an immunohistochemistry (IHC) assay. FGF23 was mainly expressed in WT osteoblast cells, which was consistent with data previously reported [8], [19]. There was a sharp increase in FGF23 expression in the *Dmp1^−/−^* osteocytes (with no FGF23 expression changes in the osteoblasts), a moderate increase in *kl/kl* osteocytes with a massive increase in *kl/kl* osteoblasts (which can be assumed as the main source for higher FGF23 serum level in *kl/kl* compared to all other groups), and a dramatic increase in *Dmp1^−/−^kl/kl* osteocytes (**Fig. 9A**). To better define the changes in FGF23 expression in osteocytes, fresh long bones (femurs) were collected, bone marrow cells were flushed out and the periosteum was removed. Real-time RT-PCR (qPCR) data confirmed a significant increase in Fgf23 mRNA in *kl/kl*, *Dmp1^−/−^* and *Dmp1^−/−^kl/kl* osteocytes with *Dmp1^−/−^kl/kl* having the highest (∼5-fold) increase (**Fig. 9B**). To address whether there was a potential linkage between the changes of FGF23 levels and apoptosis in these bones, we further examined apoptotic bone cells using the TUNEL assay. There were few detectable TUNEL positive bone cells in WT and *Dmp1^−/−^* femurs. In contrast, there was a moderate amount of apoptotic bone cells in *kl/kl* femurs and many apoptotic osteocytes in the *Dmp1^−/−^kl/kl* femurs (**Fig. 9C**). Performing quantitative analysis on osteocyte cell death showed that the apoptotic cell number in *kl/kl* mice was significantly higher than that of WT mice (**Fig. 9D**; P = 0.027), and the number of *Dmp1^−/−^* apoptotic cells was lower although with no significance (P = 0.056). The *Dmp1^−/−^kl/kl* apoptotic cells were the highest amongst all four groups (P<0.001, n = 3). Taken together, the above data showed 1) different FGF23 expression patterns in these three groups with a high level in *kl/kl* osteoblasts; 2) an anti-apoptotic role of DMP1 in a high phosphate environment; and 3) a possible correlation between a sharp up-regulation of FGF23 expression and an increase of apoptotic cell number in *Dmp1^−/−^kl/kl* osteocytes.

## Discussion

Although DMP1 is expressed in both hard and soft tissues, the phenotype in human DMP1 mutations or *Dmp1* deficient mice was mainly identified in the skeleton and the teeth, likely caused by hypophosphatemia, and increased FGF23 levels [3], [8], [27], [28]. On the other hand, defects or mutations in *Fgf23* or *Klotho* resulted in hyperphosphatemia, premature aging, and the formation of ectopic calcifications [20], [42]. To investigate the potential role of DMP1 in the *Klotho* deficient mice we generated and characterized compound deficient (*Dmp1^−/−^kl/kl)* mice using a combination of *in vitro* and *in vivo* approaches. Our studies revealed a novel anti-apoptotic function of DMP1 in a high phosphate environment (caused by *Klotho* ablation) (also see Figure S2), followed by severe ectopic calcifications within the *Dmp1^−/−^kl/kl* kidney and aorta. There is also a unique distribution of FGF23 within *kl/kl, Dmp1* ^−/−^ and *Dmp1^−/−^kl/kl* bones. These findings may stimulate future studies of the roles of DMP1 in prevention of vascular calcification in hyperphosphatemic environments such as those within chronic kidney disease (CKD).

Almost all patients with cardiovascular disease have some degree of ectopic calcification in their blood vessels and kidneys [43], although the most-extensive vascular calcification occurs in CKD patients that is closely linked to hyperphosphatemia [44]. Recent studies suggest that two key factors are responsible for vascular calcification in hyperphosphatemia: 1) up regulation of osteogenic factors such as Osterix, Cbfa1, and several bone-associated proteins (Osteopontin, Bone Sialoprotein, Alkaline Phosphatase, and Type I Collagen) [44], [45] and 2) phosphate-induced apoptosis [24], [25]. Furthermore, the recombinant Klotho protein itself is able to attenuate cellular apoptosis and senescence through mitogen-activated kinase and extracellular signal-regulated kinase pathways [46]. Here, for the first time, we showed that DMP1 plays an anti-apoptotic function in a high phosphate environment, which makes it a critical factor for the attenuation of calcification in the aorta and the kidneys in a hyperphosphatemic environment such as within CKD disease (**Fig. 7**). This anti-apoptotic role was also observed in bone (**Fig. 9C–D**), as well as in osteoblast cell culture (**Fig. S1**) when the phosphate level increased. It appears that this protection is important for maintaining osteocyte morphology in a high phosphate environment, as there was only a moderate loss of osteocyte dendrites in the *kl/kl* mice in contrast to dramatic loss of osteocyte dendrites in *Dmp1^−/−^kl/kl* mice (**Fig. 8**).

It is well documented that FGF23 is mainly produced and secreted from bone cells. However, it was debatable whether FGF23 is secreted from osteocytes or osteoblasts [6], [8], [19], [47], [48]. Here, our immunohistochemical data showed that FGF23 was mainly expressed in the osteoblast cell layer with a low level of expression in the osteocytes in both WT and *kl/kl* mice, whereas FGF23 was mainly expressed in the osteocyte in both *Dmp1* ^−/−^ and *Dmp1^−/−^kl/kl* mice (**Fig. 9A**). The qPCR data was in agreement with the above immunohistochemical data (**Fig. 9B**). Although we do not know the mechanistic details for such a difference in the sites of FGF23 expression among these animal models, the high level of FGF23 in the *Dmp1^−/−^* osteocytes was likely the consequence of the defect in the osteocyte maturation process [8], [19], [48]. In these studies, we showed that DMP1 is highly expressed in osteocytes with an extremely low level in the WT osteoblasts, and that *Dmp1^−/−^* mice displayed pathological changes in the osteocyte, including morphological changes in lacunocanalicular system, defects in matrix surrounding the osteocytes, up-regulation of many genes expressed in normal osteoblasts (such as type I collagen or BSP) or ectopically produced (such as FGF23 and osteocalcin). It appears that there was a close correlation between the high expression levels of FGF23 and the accelerated levels of apoptosis within the *Dmp1^−/−^kl/kl* osteocytes (**Fig. 9C–D** and **Fig. S2**). Our future study seeks to understand why and how apoptotic osteocytes release more FGF23 in this animal model.

*Dmp1* ^−/−^ mice, like hyp mice (a well-studied hypophosphatemic rickets animal model) [49], display abnormally high FGF23, low serum Pi, rickets and osteomalacia, which is a consequence of the defects in the osteocytes [2], [8], [19]. The administration of a high phosphate diet [8] or performing injections of FGF23 neutralizing antibody [19] can fully rescue the rickets phenotype but is only partially restored in osteomalacia. However, *Dmp1^−/−^kl/kl* mice display no sign of rickets or osteomalacia (**Fig. 4**), as these mice were never exposed to hypophosphatemia. Regarding the similarity of FGF23 and 1,25(OH)_2_D_3_ in both *Dmp1* ^−/−^ and *Dmp1^−/−^kl/kl* mice, this study does not support the direct roles of these two factors in *Dmp1* ^−/−^ bones (**Fig. 3**). Furthermore, the bone volume/total volume and osteoclast number/bone remodeling were greatly improved in the *Dmp1^−/−^kl/kl* mice, supporting the early beneficial role of Pi administration in restoring the local bone phenotype (**Figs. 2** and **5**).

Interestingly, the serum Ca level in *Dmp1^−/−^* mice is relatively “normal” compared to the age matched WT control (**Fig. 4B**), although the *Dmp1* ^−/−^ osteoclast cell number is reduced more than 50% (**Fig. 5**). This “normal Ca level” in *Dmp1* ^−/−^ mice is likely caused by an increase of renal Ca reabsorption, as the PTH level was up-regulated almost 10-fold (**Fig. 4C**).

Both *Fgf23^−/−^* and *Klotho* deficient mice share many similarities such as hyperphosphatemia, high 1,25(OH)_2_D_3_ levels, ectopic ossification in the soft tissues, and several lines of compelling evidence suggest that FGF23’s function in the regulation of systemic phosphate and vitamin D homeostasis is klotho-dependent [3], [21], [50]. However, there is a notable difference between these two types of mice: an increase in bone volume in the *Klotho* deficient tibial metaphysis (**Fig. 2**) [51], but a reduction of bone volume in *Fgf23^−/−^* tibial metaphysis [11]. Liu *et al*. showed evidence of increased Wnt signaling in *Klotho* deficient mice, suggest a unique antagonistic role against Wnt by Klotho [52]. Furthermore, the apoptotic phenomenon has not been reported in *Fgf23* ^−/−^ mice.

In conclusion, this study presented an increase of apoptosis in the *Dmp1/klotho* compound deficient mice, which is closely linked to substantial increase of ectopic ossification in the aorta and in the kidney. Discovering this novel anti-apoptotic role of DMP1 in a hyperphosphatemic environment may have important clinical relevance in uncovering a method to protect cells from apoptosis in high phosphate environments such as that which occurs in CKD patients. Our studies also showed improvement of the rickets/osteomalacia phenotype, and dramatically enhanced bone modeling in the compound deficient mice, suggesting that the low phosphate level plays a key pathological role in *Dmp1^−/−^* mice.

## Supporting Information

Figure S1 **Cell proliferation and apoptosis in osteoblasts.** As a result of protective role of DMP1 in hyperphosphatemia, increase in phosphate level increases the apoptosis level significantly in *Dmp1^−/−^* cells while it has no significant effect on WT cells *in vitro*; Primary calvarial cells were isolated from control (WT) and *Dmp1* ^−/−^ mice at age of 3-day after birth. Cells were grown in α-MEM supplemented with 10% FBS only (normal media), or added 1 or 5 mM of phosphate to the normal media. After 24 hours of incubation cells were fixed and analyzed for apoptosis or cell proliferation assay respectively. (**A**) TUNEL assay showed *Dmp1^−/−^* apoptotic cells in normal media are significantly lower than WT. Also increasing the phosphate level significantly increases the apoptosis level in *Dmp1^−/−^* cells compared to normal media. While increased phosphate level does not have any significant effect on WT cells apoptosis rate. (*P<0.05 compared to WT in normal media, §P<0.05 compared to *Dmp1^−/−^* in normal media). (**B**) Cell proliferation assay does not show any significant difference between *Dmp1^−/−^* cell proliferative activity by increasing phosphate level, confirming the protective role of DMP1 for osteoblasts in hyperphosphatemic environment. Increased phosphate level is toxic to the cells if they do not express DMP1, and increases apoptosis.
(TIF)

Figure S2 **An increase of cell death in the compound deficient bone.** (**A**) There is a dramatic increase of apoptotic cells in double deficient mice (*Dmp1* ^−/−^ *kl/kl*) compared to the single *kl/kl* osteocytes and osteoblasts in the cortical bone of femur (6 weeks old). (**B**) Hematoxylin and Eosin stain of femur cortical bone of 6 weeks old animals shows numerous empty lacunae, which represent dead osteocytes, in *Dmp1^−/−^kl/kl*. (C) Quantitative data suggest that the high empty lacunae rate in *Dmp1^−/−^kl/kl* group is significant compared to other groups (P<0.001, n = 4). Both pieces of evidence support a protective role of DMP1 in hyperphosphatemic environment.
(TIF)

Figure S3 **The** ***Dmp1*** **lacZ knock-in transgene is expressed in blood vessels.** Whole mount X-gal stain of a heterozygous *Dmp1-lacZ* knock-in uterus overnight shows blue stain blood vessel (6-mo old, *left panel*). A frozen section of this whole mount X-gal stained tissue was count stained with Eosin, displaying the blue stained cells in the blood vessel wall in small arteries, small veins and arterioles.
(TIF)

Figure S4 **The Dmp1 lacZ knock-in transgene is expressed in Kidney.** Whole mount X-gal stain of a heterozygous Dmp1-lacZ knock-in kidney overnight shows blue stain blood vessel (6-mo old). A frozen section of this whole mount X-gal stained kidney tissue was count stained with Eosin, displaying the blue stained cells in the kidney renal tubules (insert).
(TIF)
