# Supplementary figures and images for: Protective Roles of DMP1 in High Phosphate Homeostasis
Source: PLoS One. 2012 Aug 3;7(8):e42329. doi: 10.1371/journal.pone.0042329 (PMC3411740; doi:10.1371/journal.pone.0042329)

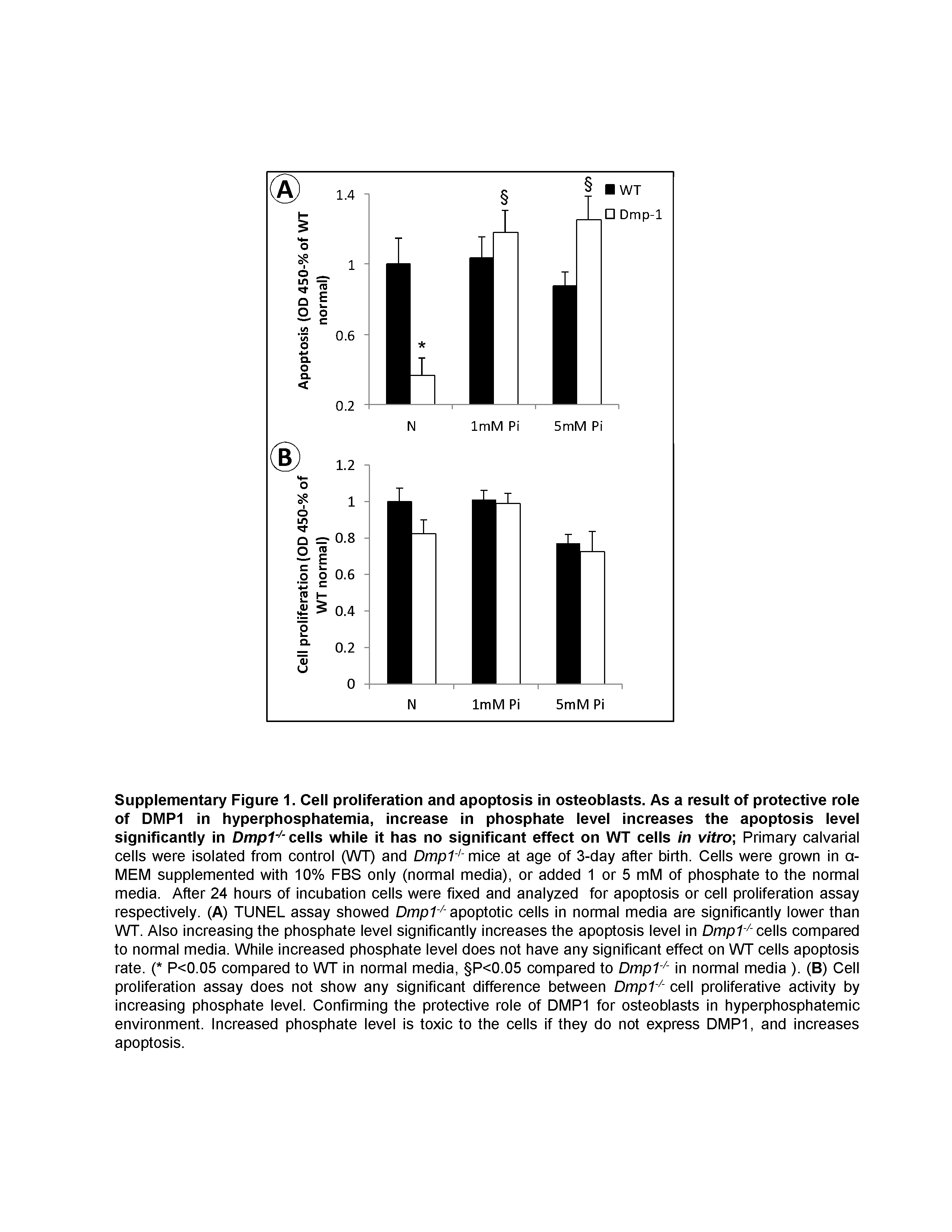

Supplement: Figure S1 — Cell proliferation and apoptosis in osteoblasts. As a result of protective role of DMP1 in hyperphosphatemia, increase in phosphate level increases the apoptosis level significantly in Dmp1−/− cells while it has no significant effect on WT cells in vitro; Primary calvarial cells were isolated from control (WT) and Dmp1 −/− mice at age of 3-day after birth. Cells were grown in α-MEM supplemented with 10% FBS only (normal media), or added 1 or 5 mM of phosphate to the normal media. After 24 hours of incubation cells were fixed and analyzed for apoptosis or cell proliferation assay respectively. (A) TUNEL assay showed Dmp1−/− apoptotic cells in normal media are significantly lower than WT. Also increasing the phosphate level significantly increases the apoptosis level in Dmp1−/− cells compared to normal media. While increased phosphate level does not have any significant effect on WT cells apoptosis rate. (*P<0.05 compared to WT in normal media, §P<0.05 compared to Dmp1−/− in normal media). (B) Cell proliferation assay does not show any significant difference between Dmp1−/− cell proliferative activity by increasing phosphate level, confirming the protective role of DMP1 for osteoblasts in hyperphosphatemic environment. Increased phosphate level is toxic to the cells if they do not express DMP1, and increases apoptosis. (TIF) [file pone.0042329.s001.tif]

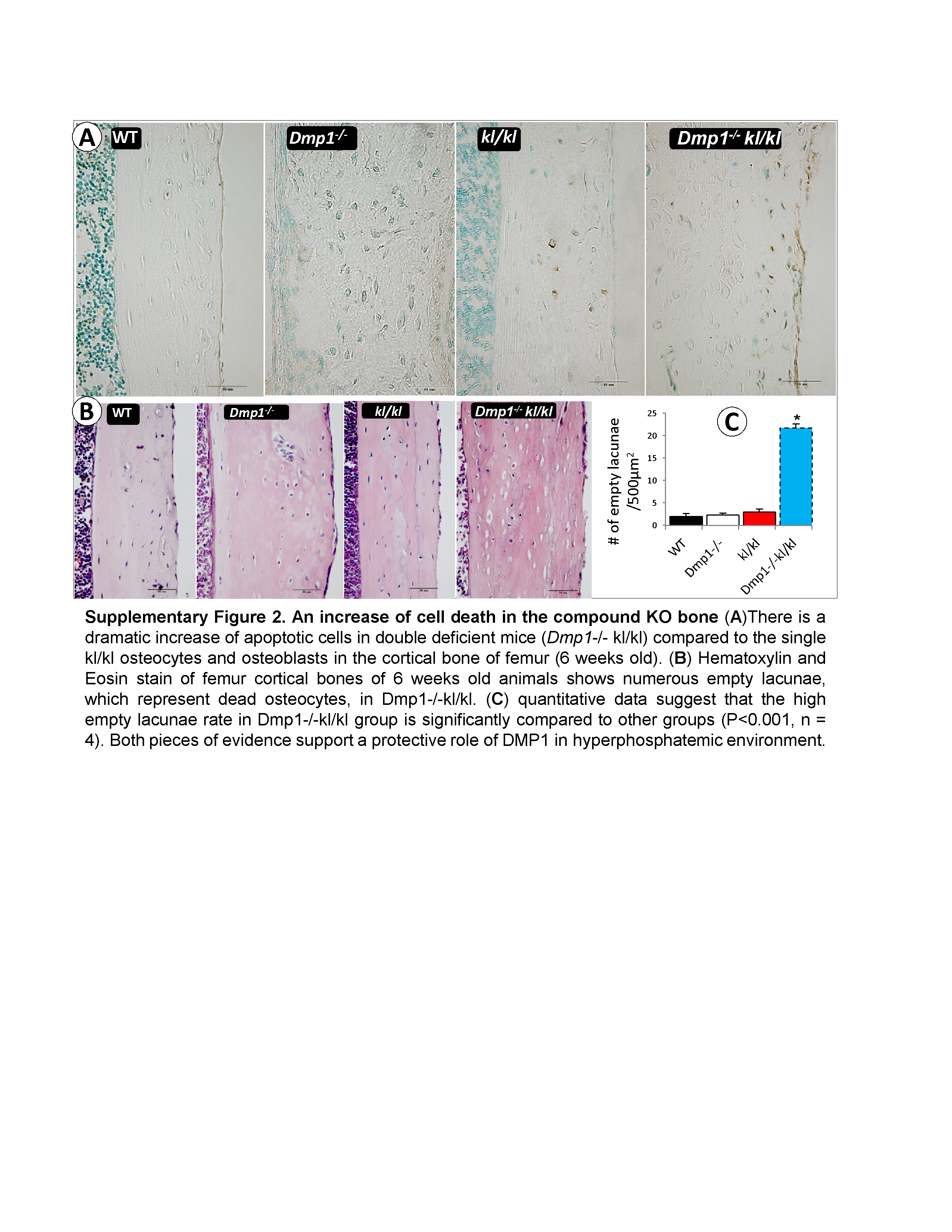

Supplement: Figure S2 — An increase of cell death in the compound deficient bone. (A) There is a dramatic increase of apoptotic cells in double deficient mice (Dmp1 −/− kl/kl) compared to the single kl/kl osteocytes and osteoblasts in the cortical bone of femur (6 weeks old). (B) Hematoxylin and Eosin stain of femur cortical bone of 6 weeks old animals shows numerous empty lacunae, which represent dead osteocytes, in Dmp1−/−kl/kl. (C) Quantitative data suggest that the high empty lacunae rate in Dmp1−/−kl/kl group is significant compared to other groups (P<0.001, n = 4). Both pieces of evidence support a protective role of DMP1 in hyperphosphatemic environment. (TIF) [file pone.0042329.s002.tif]

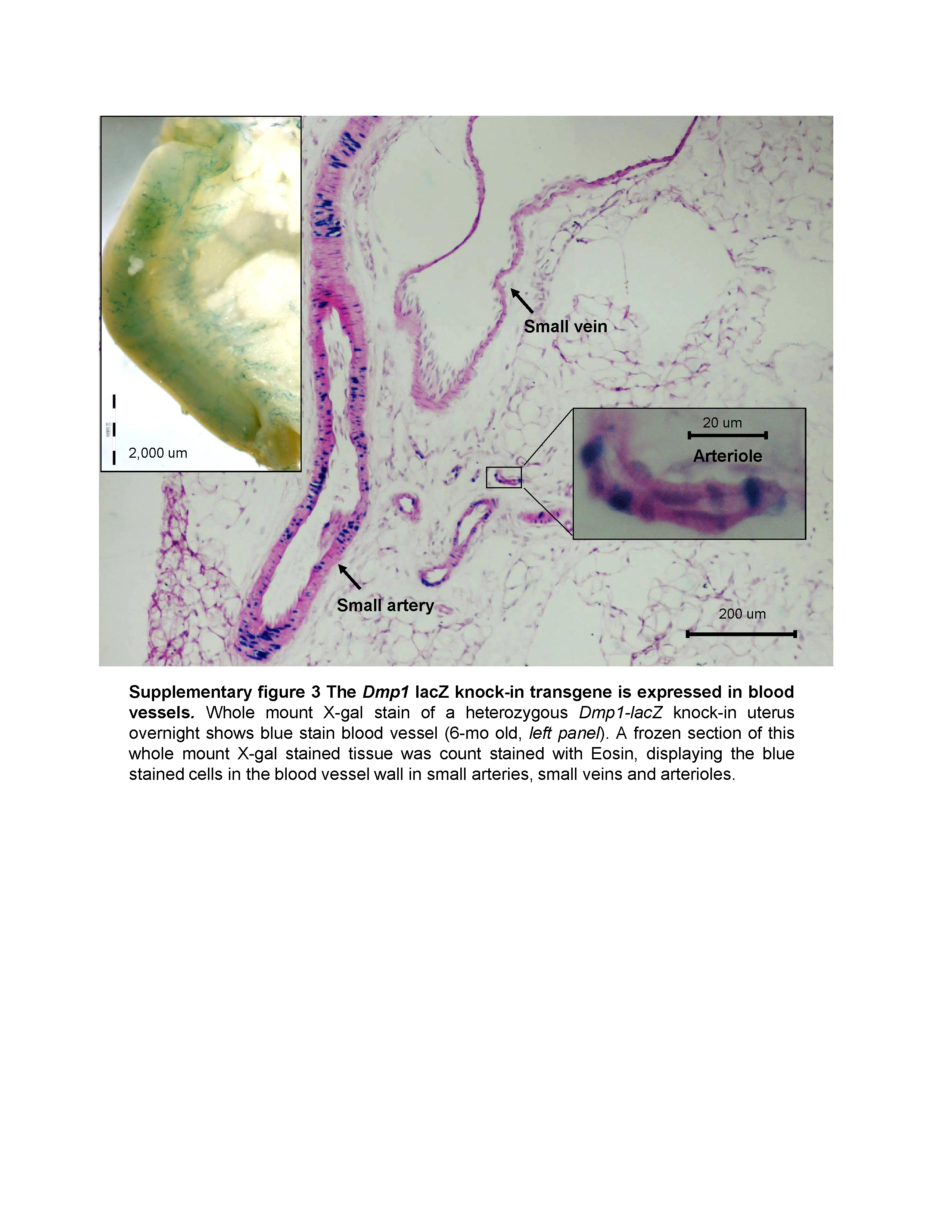

Supplement: Figure S3 — The Dmp1 lacZ knock-in transgene is expressed in blood vessels. Whole mount X-gal stain of a heterozygous Dmp1-lacZ knock-in uterus overnight shows blue stain blood vessel (6-mo old, left panel). A frozen section of this whole mount X-gal stained tissue was count stained with Eosin, displaying the blue stained cells in the blood vessel wall in small arteries, small veins and arterioles. (TIF) [file pone.0042329.s003.tif]

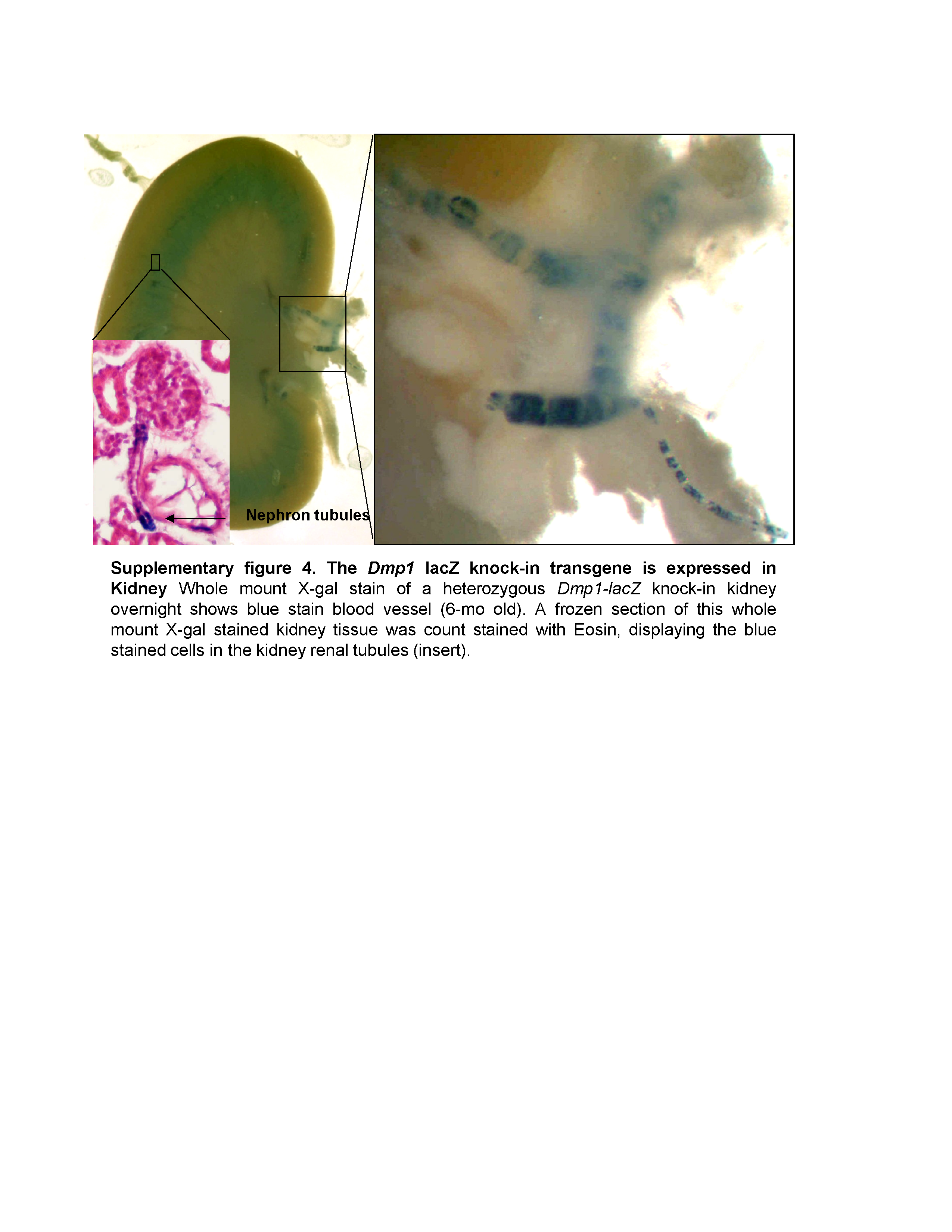

Supplement: Figure S4 — The Dmp1 lacZ knock-in transgene is expressed in Kidney. Whole mount X-gal stain of a heterozygous Dmp1-lacZ knock-in kidney overnight shows blue stain blood vessel (6-mo old). A frozen section of this whole mount X-gal stained kidney tissue was count stained with Eosin, displaying the blue stained cells in the kidney renal tubules (insert). (TIF) [file pone.0042329.s004.tif]
